# Passively harmonic dissipative soliton generation in normal dispersion erbium-doped fiber laser using SMS fiber as artificial saturable absorber

**DOI:** 10.1038/s41598-024-66111-z

**Published:** 2024-07-02

**Authors:** Yu Chen, Zian Cheak Tiu, Sin Jin Tan, Kaharudin Dimyati, Sulaiman Wadi Harun

**Affiliations:** 1 Chongqing Vocational Institute of Engineering, No.1, North and South Avenue, Binjiang New City, Jiangjin District, Chongqing, 402260 China; 2 Photonics Engineering Laboratory, Department of Electrical Engineering, Faculty of Engineering, University of Malaya, 50603 Kuala Lumpur, Malaysia; 3 Centre for Sustainable Engineering Solutions, INTI International University, 71800 Nilai, Negeri Sembilan Malaysia; 4 Faculty of Engineering, UOW Malaysia KDU University College, 40150 Shah Alam, Selangor Malaysia

**Keywords:** Optics and photonics, Optical physics

## Abstract

Passively harmonic mode-locking has been experimentally demonstrated in an erbium-doped fiber laser with large normal dispersion using single-multi-single mode structure as artificial saturable absorber. By increasing the pump power under the same polarization setting, the mode-locking operation can switch from fundamental mode-locked to 5th order harmonic mode-locked. Highest repetition rate of 4.26 MHz (5th order harmonic) is observed, with pulse width and pulse energy ascertained at 290 fs and 3.0 nJ, respectively. Excellent signal-to-noise ratio (SNR) of above 50 dB is observed for all harmonic orders. The findings validated that SMS structure can be used to generate stable and switchable high order of harmonic mode-locked. The low-cost SMS fiber for harmonic mode-locked generation technique could lay the groundwork for future sustainable industrial growth.

## Introduction

Ultrafast fiber laser with high repetition rate have been extensively investigated owing to their numerous applications in precision measurement^1^, telecommunication^2^, high-speed optical sampling^3^, biomedical treatment^4^ and optical sensing^5^. Typically, there are several ways to achieve a high pulse repetition rate. The direct method is by shortening the cavity length, which usually brings difficulties to the design of cavity. Alternatively, active modulation is another solution to obtain high repetition rate pulses. However, active modulation required a complex external system to achieve the desired output. In all-fiber laser systems, harmonic mode-locked (HML) is the commonly adopted approach to achieve high pulse repetition rate. By adopted HML approach, the constraint of gain medium length is no longer a limiting factor.

The use of saturable absorber (SA), be it real or artificial SA is an important technique that has been extensively explored for passively mode-locked in a fiber laser system^6–8^. It synchronizes and locks the phase of oscillating light in a cavity with a coherent manner. This eventually leads to the formation of stable and narrow pulse after many round trips of absorption and transmission. Popular emerging materials SA for HML include single-walled carbon nanotubes (SWCNTs)^9,10^, topological insulator (TI)^11,12^, transition metal dichalcogenide (TMD)^13,14^, graphene^15,16^ and MXene^17,18^. Above emerging materials offer high optical absorption and fast recovery time that favorable for mode-locking operation. However, they usually suffer from low damage threshold, and complex fabrication process. On contrary, artificial SAs such as nonlinear polarization rotation (NPR)^19–21^, nonlinear amplifying loop mirror (NALM)^22^ and nonlinear multimode interference (NL-MMI)^23^ are adopted to tackle the challenges associated with emerging material SA, while providing similar mode-locking effect. HML with artificial SA is simple as it involves manipulation of light using all-optical components. Recently, NL-MMI technique utilizing the single-multi-single mode (SMS) where the multimode section is made from graded index multi-mode fiber (GIMF) has garnered the attention of researchers. The SMS fiber exhibits high thermal damage threshold, and it enhances nonlinearity of the fiber laser cavity. These are the desired properties to facilitate stable HML generation in fiber laser system. GIMF is widely studied in the nonlinear optical interactions scope, as the group velocities of all modes in GIMF are nearly identical at specific wavelengths. Thus, the use of GIMF as multimode section further enhances the feasibility of mode-locking operation using SMS structure.

In this work, we report HML in an erbium-doped fiber laser (EDFL) with large normal dispersion using SMS structure as artificial SA. The repetition rate of the pulses can be tuned from 858 kHz to 4.29 MHz (5th order harmonics) by increasing the pump power under the same polarization setting. At the 5th order harmonics, the highest pulse energy was 3.0 nJ at 4.26 MHz, and the pulse width is measured at 290 fs. The signal-to-noise ratio (SNR) is above 50 dB for all the harmonics orders, signifying its excellent stability. It is worth emphasizing that the stability mode-locking operation is initiated at a very low threshold in this work.

## Experimental setup

**Figure 1:**
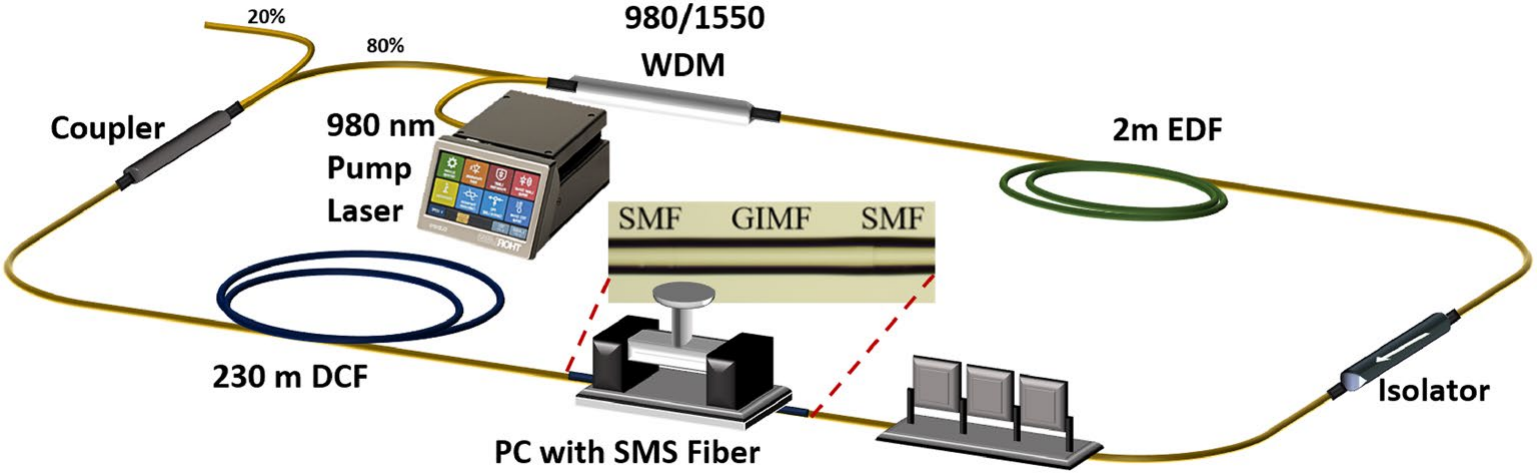
Cavity setup of HML EDFL using SMS as SA.

Figure 1 depicts the experimental setup for the proposed HML EDFL using SMS as artificial SA. A 980 nm pump laser triggers the excitation at the EDF through a wavelength division multiplexer (WDM). The function of an isolator (ISO) is to make sure the laser is circulating unidirectionally in the cavity. A 3 paddles PC is used to manipulate the polarization state of the light. The 80:20 coupler completes the ring cavity by looping 80% of circulation light back to cavity, whereas another 20% of light is tapped to another 50:50 coupler for further analysis. The optical spectrum of mode-locked laser is analyzed using optical spectrum analyzer (OSA, Yokogawa AQ6370D). The temporal characteristics are visualized using a photodetector (EOT, ET-3500F) and oscilloscope (GW Instek GDS-3352). Output power is measured by an optical power meter (Thorlabs, PM100D), while the pulse width measurement is carried out using an autocorrelator (Alnair Labs HAC-200). The stability of the pulse is ascertained using RF spectrum analyzer (Anritsu, MS2830A). The dispersion coefficient of EDF and DCF is − 13.1 ps/nm∙km and − 4.0 ps/nm∙km, respectively. The total cavity length is approximately 239.5 m with total net dispersion of 1.07 ps^2^. The EDF laser is operating at a large normal dispersion. The SMS is constructed using a 10.0 cm length of GIMF (YOFC, OM4) which is fusion spliced (Fujikura, 90S +) in between two SMF fibers to form the SMS structure and placed in an inline PC. The inline PC is used to hold the GIMF straight, and to apply stress on the GIMF. The applied stress is favorable to micro-adjust the propagation path of the light and achieved the optimized self-imaging distance. The splicing loss of SMF-GIMF at both ends are 0.01 dB and 0.02 dB respectively. The standard SMF has core/cladding dimension of 9/125 µm, whereas the GIMF has a core/cladding dimension of 50/125 µm. The nonlinear transmittance of SMS fiber has attached as Fig. I in supplementary material. When we add a PC before the SMS fiber, the nonlinear transmittance varies with the change of polarization state. This could be an interesting future study to investigate the relevancy of nonlinear transmittance between the polarization state with SMS fiber.

## Results and discussion

The length of GIMF is crucial in the order of self-imaging distance. When light propagates from SMF to GIMF, low power signal encounters higher loss due to core diameter mismatch between SMF and GIMF. At the same time, it triggers the excitation of higher order modes. When the light reaches half the self-imaging distance, low power signal may experience significant loss. At self-imaging distance, constructive interference takes place and allows higher power of light to recombine. This process repeats along the GIMF, and it can differentiate high and low light intensity, and efficiently couples high power signal into the next section of SMF. The relationship between Kerr effect and the self-imaging distance is described by,

1 $$\Delta {\eta }_{eff,n}\left(I\right)L={m}_{0}{\lambda }_{0}, $$

where $\Delta {\eta }_{eff,n}\left(I\right)$ is the effective refractive index difference for propagating modes at different intensity, $L$ is the self-imaging distance, ${m}_{0}$ represents the order of self-imaging while ${\lambda }_{0}$ describes the wavelength of light^24^.

**Figure 2:**
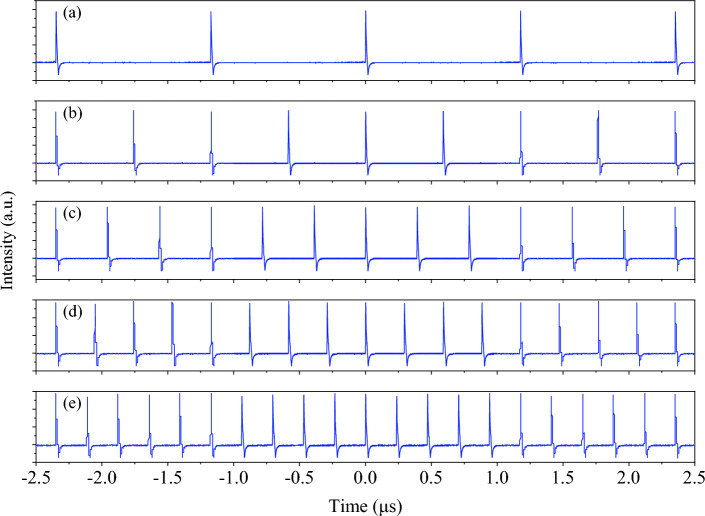
Pulse trains of the laser (**a**) FML at 858 kHz, pump power 23.2 mW, (**b**) 2nd HML at 1.72 MHz, pump power 36.0 mW, (**c**) 3rd HML at 2.56 MHz, pump power 53.9 mW, (**d**) 4th HML at 3.40 MHz, 73.4 mW, and (**e**) 5th HML at 4.29 MHz, pump power 97.0 mW.

**Figure 3:**
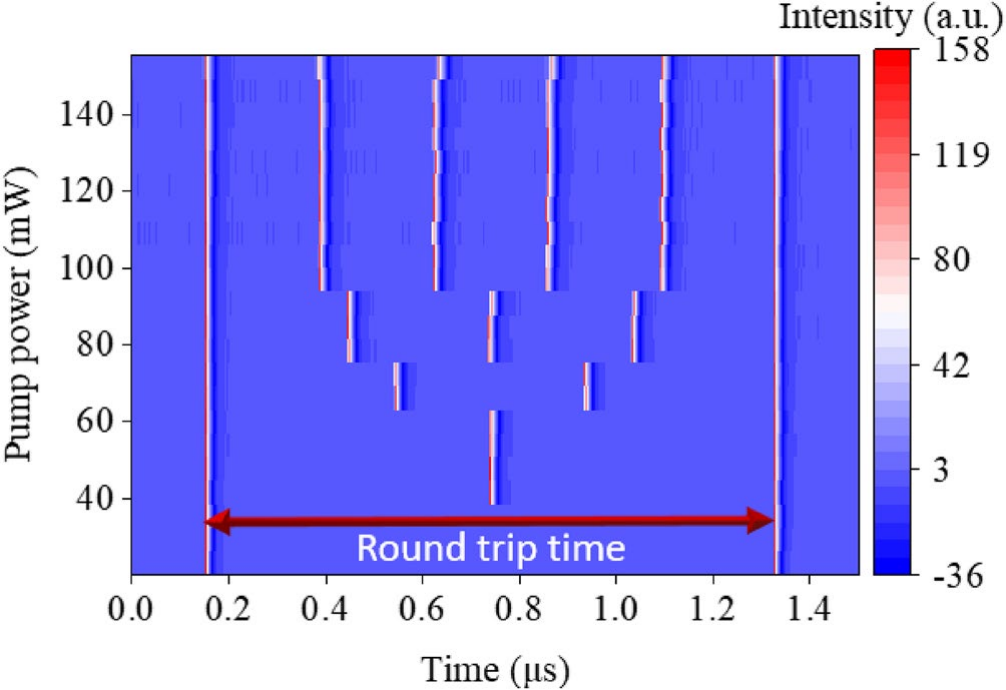
2D contour view of pulse train at various pump power.

With the pump power gradually increased to 23.2 mW, mode-locking operation is self-started. The variance of core diameter between SMF and MMF promotes multimode interference and coupling of high-power signals, effectively simulating an SA. The optimal length of GIMF, together with the low loss cavity contributed to the low threshold power to initiate mode-locking. The repetition rate is revealed at fundamental mode-locking of 858 kHz, and it corresponded with the total cavity length of 239.5 m. When the pump power further increased to 36 mW, the repetition rate doubled to 1.72 MHz, and it regarded as 2nd harmonic mode-locked. The 3rd (2.56 MHz) and 4th (3.40 MHz) harmonic mode-locked commenced at pump power of 53.9 mW and 73.4 mW, respectively. Once the pump power touches 97 mW, the repetition rate changes from 3.40 to 4.29 MHz. As the pump power is further increased up to the maximum available pump power, 5th harmonic mode-locking remained. When pump power is raised, the pulse intensity circulating in the cavity increases along. However, the increase of pulse intensity is limited due to gain depletion in the laser cavity. The clamping of the pulse modulates the intensity of pulse train, leading to the periodic modulation behavior at multiples of the fundamental frequency, and equally distributed. Additionally, the nonlinear phase modulation induced GIMF can effectively phase-lock the pulses at multiple of the fundamental repetition rate. The evolution of pulse train in temporal domain from fundamental frequency to 5^th^ harmonic is represented in Fig. 2. When the pump power increased, the harmonic order evolved accordingly, and led to the increase of pulses within a round trip time. This could visualize better in the contour view of temporal domain evolution against pump power as shown in Fig. 3.

**Figure 4:**
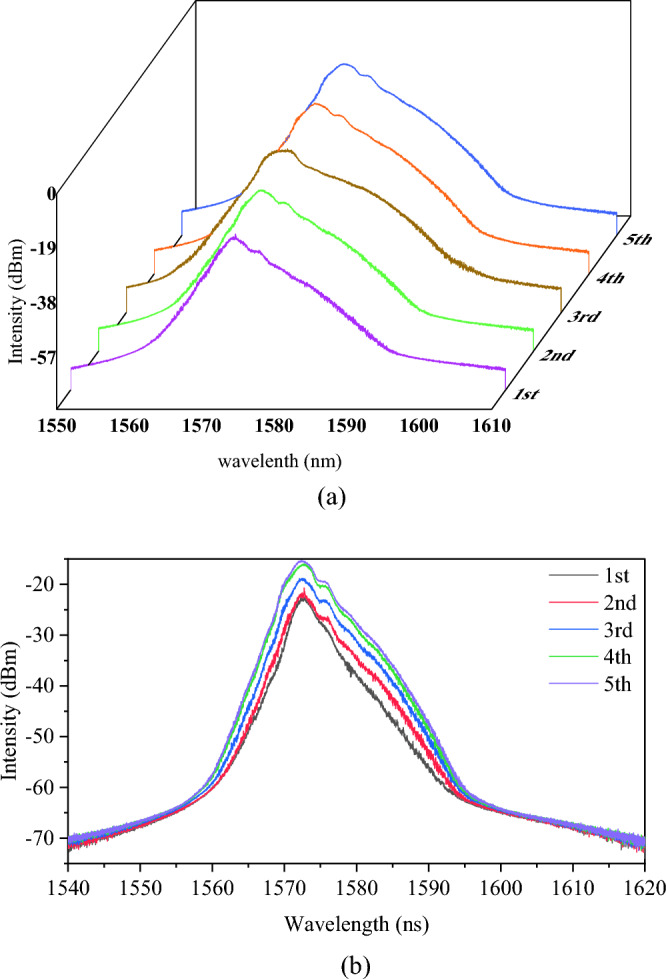
The optical spectrum of different at different harmonic orders at (**a**) 3D view and (**b**) 2D view.

**Figure 5:**
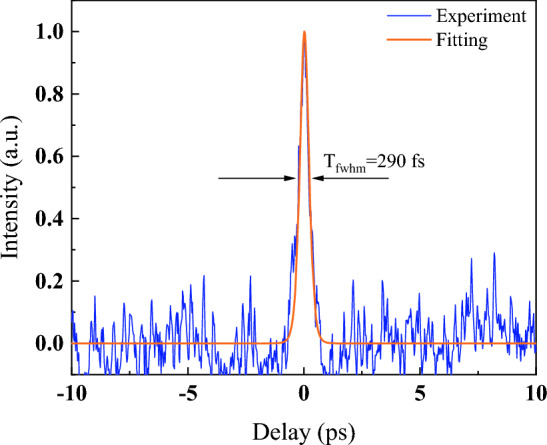
The autocorrelation trace of the pulse at 5th harmonic.

**Figure 6:**
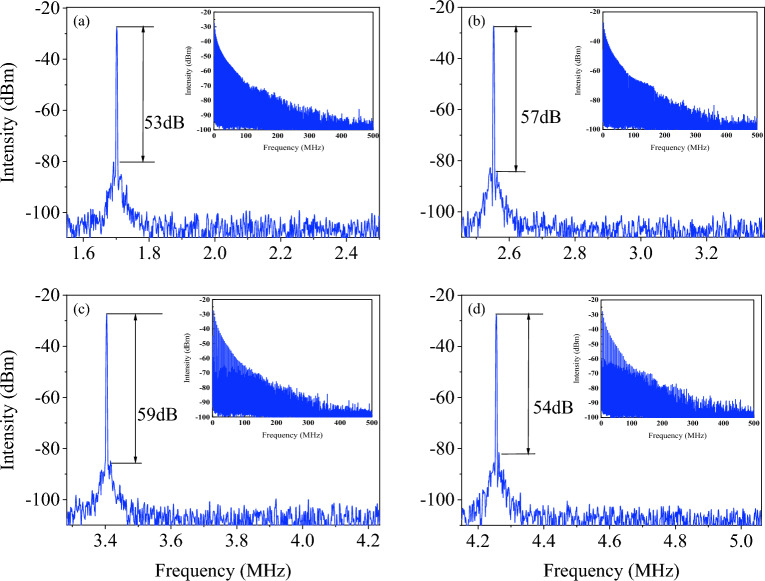
RF spectrum of (**a**) 2nd HML at 1.72 MHz, (**b**) 3rd HML at 2.56 MHz, (**c**) 4th HML at 3.40 MHz, and (**d**) 5th HML at 4.29 MHz.

**Figure 7:**
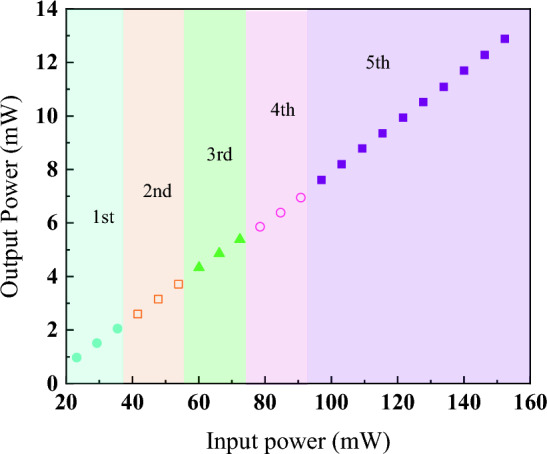
Output power vs. input power at different harmonic orders.

The corresponding optical spectrum with the harmonic order is illustrated in Fig. 4. The peak power increases while the spectrum broadens with pump power. The peak power increases from − 17.96 dBm (FML) to − 15.25 dBm (5th harmonic), while maintaining the center wavelength at 1572 nm. Figure 5 illustrates the pulse width obtained at 5th harmonic order. The estimated pulse width is 290 fs using Gaussian curve fitting. The narrow pulse width achieved can be attributed to the dispersion compensation effect provided by DCF. Additionally, the pulse width at fundamental mode-locked is measured as 430 fs, which is comparable to previous work^25^. With the increase of harmonic order, the pulse width achieved 290 fs at 5th harmonic order, indicating the pulse compression happened at higher harmonic order^26^. Furthermore, the cavity output is connected to the autocorrelator using a 200 m SMF, which could trigger pulse compression outside the cavity^27,28^. The 200 m SMF used for the connection between cavity and autocorrelator is due to the facility layout, and it is not an optimized length for pulse compression. It required further in-depth analysis to pave the details of pulse compression and achieve the optimized results, which could be a feasible extension work from the current results. Figure 6 shows the signal-to-noise (SNR) ratio of the harmonic pulses. The SNR kept above 50 dB for all the harmonic orders, which indicated the switching of harmonic orders are highly stable. Insets show the broader span of frequency spectrum up to 500 MHz. No obvious spectral modulation is observed, which further validated the stability for all harmonic orders. However, by taking supermode noise into account, the SNR for 2nd, 3rd, 4th, and 5th harmonic orders are dropped to 32 dB, 31 dB, 35 dB and 34 dB respectively as shown in Fig. II in supplementary material. The output power throughout the mode-locking operation is traced as depicted in Fig. 7. The output power increases with pump power, and the maximum output power is recorded as 12.9 mW. The corresponding pulse energy is 3.0 nJ. Harmonics order beyond 5th harmonics is not observed within the available pump power. It could probably be due to saturation of gain medium. The increment of pump power is not proportional to the increase in gain. Thus, it cannot amplify the pulse to higher harmonic order efficiently. Table 1 compiles the previous works on HML with SMS. Owing to the micro-adjustment of the light propagation path using inline PC, the threshold power for mode-locking operation in this work is relatively lower than other works. Additionally, the high order harmonic pulse compression assisted the reported pulse width to outperform as shown in Table 1. However, the mechanically controlled PC to optimize the mode-locked performance could be a potential constraint for the reported mechanism to commercialize in near future.

**Table 1:** Comparison of HML in fiber laser using SMS as SA.

Structure	Threshold pump power	Operating wavelength	Pulse duration	Repetition rate	Harmonic order	Refs.
SMF-SIMF-GIMF-SMF	490 mW	1024.7 nm,	360 fs	103.3 MHz	3	^29^
SMF-GIMF-SMF	90 mW	1558.38 nm	1.52 ps	286 MHz	16	^23^
SMF-MMF-SMF	81 mW	1560.15 nm	1.02 ps	1.26 GHz	22	^30^
SMF-NCF-GIMF-SMF	180 mW	1895.7 nm	1.25 ps	587.5 MHz	31	^31^
SMF-FMF-SMF	210 mW	1878.2 nm	1.98 ps	100.7 MHz	12	^32^
SMF-GIMF-SMF	492 mW	1558 nm	4 ps	45.28 MHz	8	^33^
SMF-GIMF-SMF	23.2 mW	1572 nm	290 fs	4.29 MHz	5	This work

## Conclusion

In conclusion, passively HML is demonstrated in an EDFL using SMS fiber as artificial SA. By increasing the pump power, FML evolves progressively to 5th harmonic order, with 5th order repetition rate of 4.29 MHz. The pulse width and pulse energy at 5th harmonic is ascertained at 290 fs and 3.0 nJ, respectively. The SNRs are kept above 50 dB, demonstrating the high stability of the HMLs. This work shows that SMS structure as saturable absorber can generate stable harmonics in normal dispersion regime. For future work, the cavity design can be further optimized to avoid gain saturation so that higher harmonic orders can be generated.

### Supplementary Information

Supplementary Figures.

## Data Availability

The datasets used and analyzed during the current study are available from the corresponding author on reasonable request.
