# Supplementary material for: Passively harmonic dissipative soliton generation in normal dispersion erbium-doped fiber laser using SMS fiber as artificial saturable absorber
Source: Sci Rep. 2024 Jul 2;14:15134. doi: 10.1038/s41598-024-66111-z (PMC11220008; doi:10.1038/s41598-024-66111-z)
Supplement: Supplementary file 1 — Supplementary Figures. [file 41598_2024_66111_MOESM1_ESM.docx]

**Supplementary Materials**


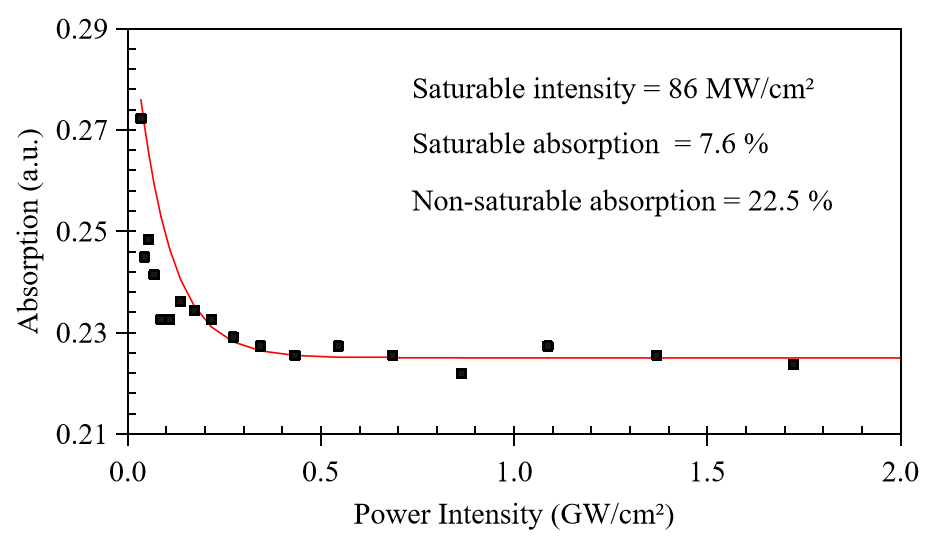


Figure I: Nonlinear transmittance of SMS fiber.

Figure II: SNR with the consideration of supermode noise for (a) 2^nd^ harmonic, (b) 3^rd^ harmonic, (c) 4^th^ harmonic and (d) 5^th^ harmonic orders.
